# MITOCHONDRIAL SUBSTRATE UTILIZATION FOLLOWING CONTRACTION IN 3D ENGINEERED MUSCLE TISSUE MODELS OF AGING

**DOI:** 10.1093/geroni/igae098.2441

**Published:** 2024-12-31

**Authors:** Matthew Campbell, Jayma Erker, David Mack, David Marcinek

**Affiliations:** University of Washington, Seattle, Washington, United States; University of Washington, Seattle, Washington, United States; University of Washington, Seattle, Washington, United States; University of Washington, Seattle, Washington, United States

## Abstract

Altered mitochondrial function in aging skeletal muscle leads to elevated oxidative stress, reduced substrate oxidation and energy maintenance that contribute to decreased skeletal muscle mass, specific force and increased fatty infiltration and frailty with age. Metabolic flexibility in response to increased energy demand is a critical component of the mitochondrial adaptive response to muscle contraction. We have found that aged muscle mitochondria do not utilize substrates the same way young muscle mitochondria do following high-intensity and low-intensity contraction. We also found that high-intensity contraction specifically inhibits oxidation of glutamate in both stimulated and non-stimulated aged muscle, suggesting that intense muscle contraction in aged muscle may inhibit substrate flexibility beyond the contracting myofibers. To test the mechanisms that contribute to this impaired metabolic signaling to muscle contraction in a human cell model of aging we have partnered with the Study of Muscle, Mobility and Aging to obtain primary human myoblasts from well phenotyped older adults to develop a three-dimensional engineered muscle tissue (3D-EMT) model of skeletal muscle aging. 3D-EMT force and contraction/relaxation kinetics were tested with high reproducibility using a Magnetometric Analyzer for eNgineered Tissue ARRAY (Mantarray, Curi Bio) platform. Aged 3D-EMT shows characteristic force-frequency and increased force up to 28 days after tissue casting and increased mitochondrial oxidative phosphorylation following contraction. Developments in tissue engineering using primary cells purified directly from patients offer some of the best opportunities yet to link specific mechanistic tests of metabolic and muscle function to patient data.

